# Adult ADHD as a risk factor for dementia: integrating longitudinal evidence, mechanistic insights, and the role of stimulant treatment

**DOI:** 10.3389/frdem.2025.1735357

**Published:** 2025-12-16

**Authors:** Angel Golimstok, Waleska Berrios

**Affiliations:** 1 Universidad Hospital Italiano de Buenos Aires, Buenos Aires, Argentina; 2 Department of Neurology, Hospital Italiano de Buenos Aires, Buenos Aires, Argentina

**Keywords:** adult attention-deficit/hyperactivity disorder, biomarkers, cognitive reserve, Lewy body disease, Wnt/mTOR pathway, dementia

## Abstract

Adult attention-deficit/hyperactivity disorder (ADHD) is increasingly recognized as a persistent neurodevelopmental condition with long-term implications for cognitive aging and dementia risk. Epidemiological and longitudinal studies indicate that adults with ADHD have a higher incidence of non-amnestic dementia subtypes, including Lewy body disease, and may be particularly susceptible to age-related cognitive decline. Convergent mechanistic evidence implicates dopaminergic dysregulation, possible Wnt/mTOR pathway alterations, oxidative stress, and chronic neuroinflammation as shared biological pathways linking ADHD to neurodegeneration. Psychiatric comorbidities, lifestyle factors, and reduced cognitive reserve may further amplify vulnerability, decreasing the brain’s resilience to neuropathological insults. Preliminary data suggest that stimulant treatments, such as methylphenidate and amphetamine derivatives, may attenuate dementia risk by normalizing dopaminergic tone, enhancing cortical plasticity, and reducing oxidative stress, although randomized trials are needed to confirm these protective effects. Emerging biomarker and genetic studies, including polygenic ADHD risk scores, α-synuclein, amyloid/tau, and GBA mutations, offer opportunities for early detection, risk stratification, and mechanistic insights. Integrating epidemiological, neuroimaging, molecular, and pharmacological data could guide targeted preventive interventions, highlighting ADHD as a developmental and potentially modifiable risk factor for late-life neurodegenerative disorders.

## 1 Introduction

Attention-deficit/hyperactivity disorder (ADHD), once considered a childhood-limited condition, is now well established as a persistent neurodevelopmental disorder that often continues into adulthood (Faraone et al., 2021). Adult ADHD affects approximately 2.5–3.5% of the global population and is commonly associated with psychiatric disorders, cardiovascular conditions, metabolic syndrome, and substance use (Cortese, 2012). Adult ADHD is recognized as a clinically significant entity with profound consequences for academic achievement, occupational functioning, interpersonal relationships, and overall quality of life (French et al., 2024). Beyond these psychosocial effects, emerging evidence suggests that ADHD in adulthood may have long-term implications for brain health and cognitive aging, raising questions about its role as a potential risk factor for dementia (Golimstok et al., 2011; Fluegge and Fluegge, 2018; Tzeng et al., 2019; Dobrosavljevic et al., 2021; Zhang et al., 2022; Levine et al., 2023). Recent evidence further extends this perspective by demonstrating that ADHD symptoms not only persist in older age but also retain a structured phenotype characterized by inattention, impulsivity, and hyperactivity, with inattention being most prominent. These findings, derived from large-scale population-based data, emphasize the clinical relevance of ADHD well beyond midlife (Levine et al., 2025).

There is growing evidence that ADHD and mild cognitive impairment (MCI) share early neuropsychological deficits, especially in areas such as attention, working memory, and executive functioning, which may reflect overlapping mechanisms (Callahan et al., 2022). According to the existing publications on the subject, a paradigm shift has occurred over the past decade. ADHD is increasingly recognized not only as a disorder of attention and impulse control, but also as a possible long-term risk factor for neurodegenerative diseases. Concurrently, several studies were released analyzing the relationship between ADHD, cognitive impairment, and dementia.

This narrative review aims to synthesize emerging longitudinal and mechanistic evidence linking ADHD with increased risk of neurodegenerative diseases, particularly dementia. Despite growing epidemiological data, the underlying biological mechanisms remain poorly delineated, and no unified framework currently integrates neurodevelopmental, molecular, and treatment-related pathways. Additionally, the potential modulating role of cognitive reserve in this association has received limited attention. This work intends to address these gaps by proposing an integrative framework that bridges the fields of developmental neurobiology and late-life neurodegeneration.

## 2 Methods

In this narrative review, an electronic literature search was conducted using major biomedical databases, including MEDLINE/PubMed, Scopus, and Google Scholar, to identify relevant publications from 2009 to October 2025. This time frame was selected because the conceptual link between adult ADHD and neurodegenerative diseases began to emerge more clearly in the late 2000s, leading to a progressive expansion of empirical and theoretical work in subsequent years. The search focused on original research articles, systematic reviews/meta-analyses, and theoretical contributions addressing aspects of the association between adult ADHD and neurodegeneration.

Both controlled vocabulary and free-text terms were used in a flexible combination, including: “adult ADHD,” “dementia,” “neurodegeneration,” “Lewy body disease,” “cognitive decline,” “cognitive reserve,” “Wnt signaling,” “mTOR pathway,” “dopamine,” “neuroinflammation,” “oxidative stress,” and “psychostimulant.” The reference lists of selected articles were also reviewed to identify additional relevant literature. Studies were selected based on conceptual relevance, methodological rigor, and their contribution to the integrative objectives of the review. No restrictions were imposed concerning country, sample characteristics, or study design, and literature published in both English and Spanish was considered.

## 3 Adult ADHD as a risk factor for dementia

### 3.1 Longitudinal and epidemiological evidence

Becker et al. (2023) critically reviewed existing studies on the potential link between adult ADHD and later neurodegenerative disease, particularly Lewy body disorders. They found that while several studies report an increased risk of dementia among individuals with ADHD, methodological limitations, such as diagnostic inaccuracy and sample heterogeneity, make current evidence inconclusive. The authors emphasized the need for well-designed prospective studies to clarify whether ADHD represents an independent risk factor for neurodegeneration (Becker et al., 2023). Dobrosavljevic et al. (2021) published a large retrospective cohort study using Swedish national registers, including 3.6 million individuals born between 1932 and 1963. Based on ICD diagnoses and prescription data, adults with ADHD showed an increased risk of dementia (HR = 2.92, 95% CI 2.40–3.57) and MCI (HR = 6.21, 95% CI 5.25–7.35). After adjusting for psychiatric comorbidities, these associations were notably attenuated (HR = 1.62 and 2.54, respectively). The authors concluded that ADHD may heighten vulnerability to cognitive decline, although shared psychiatric factors likely contribute, underscoring the need for prospective research to clarify causality (Dobrosavljevic et al., 2021).

Recent prospective cohort studies have addressed the association between adult ADHD and subsequent neurodegenerative diseases, aligning with the need for well-designed research highlighted by Becker et al. (2023). Levine et al. (2023) published a nationwide Israeli cohort of 109,218 adults (ages 51–70 at baseline) followed for up to ~17 years, who were free of ADHD and dementia diagnoses at baseline. The study found that adult ADHD was associated with a 2.77-fold increased risk of dementia (95% CI 2.11–3.63) after adjusting for 18 potential confounders. Notably, no increased risk was observed among those treated with psychostimulant medications (Levine et al., 2023). Although this finding could suggest a potential protective effect of treatment, further investigation is required. The authors emphasized the importance of recognizing adult ADHD as a potential risk factor for dementia. They highlighted the need for clinicians to monitor individuals with ADHD for cognitive decline and consider early interventions, suggesting future research should focus on understanding the underlying mechanisms linking ADHD to dementia and exploring the long-term effects of ADHD treatments on cognitive health.

Another prospective cohort study conducted in Argentina followed participants diagnosed with ADHD in adulthood over a 15-year period (Golimstok et al., 2024). The study found that adult ADHD was independently associated with an increased risk of developing Lewy body disease (LBD), dementia, and non-amnestic MCI. These findings underscore the importance of monitoring cognitive health in individuals with a history of adult ADHD. That publication discussed the potential pathophysiological mechanisms linking ADHD to neurodegenerative diseases, including dopaminergic dysfunction and neuroinflammation. The authors called for further studies to elucidate these mechanisms and to determine whether early treatment of ADHD could mitigate the risk of developing neurodegenerative diseases later in life.

Collectively, these longitudinal and cohort studies strengthen the epidemiological link between adult ADHD and subsequent neurodegenerative risk, fulfilling the methodological criteria that the earlier reviews mentioned above had identified as necessary. However, while these findings emphasize a reproducible association, the underlying biological mechanisms remain incompletely understood. Converging neuroimaging, molecular, and clinical evidence suggests that ADHD and neurodegenerative disorders such as Alzheimer’s disease (AD) and Lewy body dementia may share common neurobiological pathways involving dopaminergic imbalance, neuroinflammation, oxidative stress, and signaling pathway dysregulation. Understanding these mechanistic intersections is critical to explain how a developmental disorder like ADHD could evolve into, or predispose toward, neurodegenerative vulnerability in later life.

The following section summarizes current mechanistic hypotheses integrating neurobiological, molecular, and comorbidity-related processes that may mediate the observed epidemiological association.

### 3.2 Mechanistic pathways: neurobiology and comorbidities

#### 3.2.1 Dopaminergic dysfunction in ADHD and its relevance to neurodegenerative diseases

Evidence from brain imaging, genetic, and pharmacological studies supports that ADHD involves dopaminergic dysregulation within mesocortical and mesostriatal circuits, including the prefrontal cortex and striatum, which are critical for executive control, attention, and reward processing (Volkow et al., 2009; Véronneau-Veilleux et al., 2022; MacDonald et al., 2024). However, functional imaging and computational modeling studies indicate that ADHD does not reflect a uniform dopaminergic hypofunction but rather a tonic–phasic imbalance, characterized by reduced tonic dopamine levels and increased phasic fluctuations in cortico-striatal loops during reinforcement learning (Tripp and Wickens, 2009; Véronneau-Veilleux et al., 2022).

This concept aligns with PET and fMRI evidence showing lower D2/D3 receptor and dopamine transporter (DAT) availability in mesoaccumbens and midbrain regions, correlating with deficits in attentional control and reward sensitivity (Volkow et al., 2009; Kollins and Adcock, 2014). In contrast, Lewy body dementia and Parkinsonian dementias are characterized by progressive degeneration of nigrostriatal dopaminergic neurons in the substantia nigra pars compacta, leading to striatal dopamine loss and manifesting in motor and cognitive impairment (Lee et al., 2023; Prasad et al., 2023). This neurodegenerative pathology fundamentally differs from the functional dysregulation observed in ADHD, which is primarily neurotransmission-based rather than neurodegenerative in nature (Prasad et al., 2023).

Although ADHD is primarily functional rather than degenerative, chronic dopaminergic imbalance may lead to compensatory stress on synapses, promoting vulnerability to later neurodegenerative changes (Volkow et al., 2009).

It is noteworthy that dopaminergic abnormalities are not unique to Lewy body disorders. Increasing evidence implicates dopamine dysfunction in AD as well, where altered dopamine synthesis, receptor expression, and terminal integrity contribute to executive dysfunction, apathy, and accelerated cognitive decline. These convergent findings imply that ADHD-related dopaminergic vulnerability could interact with Alzheimer’s-relevant pathophysiology (e.g., synaptic failure, network disconnection), providing a plausible mechanistic route by which developmental dopaminergic dysregulation increases later-life risk across multiple dementia syndromes rather than only LBD (Martorana and Koch, 2014; Pan et al., 2019; Grünblatt et al., 2023).

Interestingly, previous reports have explored potential links between ADHD and Parkinson’s disease (PD), yet the evidence remains limited and inconclusive, with most studies failing to demonstrate a robust association (Becker et al., 2023). Genetic findings align with this view: Geissler et al. (2017) analyzed nine ADHD-related candidate single-nucleotide polymorphisms (SNPs) across seven genes and found no significant association with PD after correction for multiple testing. In contrast, longitudinal data now consistently indicate a stronger association between adult ADHD and dementia, particularly Lewy body dementia (Golimstok et al., 2024). Furthermore, it is important to note the high comorbidity between Lewy body dementia and AD, with many patients exhibiting overlapping neuropathological features (Galvin, 2024). This convergence supports the notion that ADHD may act as a neurodevelopmental vulnerability factor for late-life dementia, particularly in individuals at risk for mixed or overlapping Lewy body dementia –AD pathology, rather than for isolated Parkinsonian syndromes.

#### 3.2.2 Wnt/mTOR pathway dysregulation

Grünblatt et al. (2023) proposed the Wnt/mTOR hypothesis, suggesting that ADHD-related neurodevelopmental dysregulation intersects with mechanisms implicated in AD. Disruption in Wnt signaling impairs neurogenesis, synaptic plasticity, and mitochondrial function, while aberrant mTOR activation contributes to oxidative stress and protein aggregation, potentially bridging early ADHD phenotypes with late-life cognitive decline (Grünblatt et al., 2023). While most mechanistic attention has focused on the Wnt/mTOR axis in AD, emerging evidence suggests that this pathway may also contribute to the pathophysiology of other dementias. The mTOR signalling cascade (mTORC1/2) is implicated in neurodegenerative mechanisms across multiple disorders, including frontotemporal dementia (FTD) and PD, where it modulates autophagy, protein aggregation, and synaptic plasticity (Zhu et al., 2019; Querfurth and Lee, 2021). In cellular and animal models of progranulin-deficient frontotemporal lobar degeneration (FTLD-TDP), both canonical and non-canonical Wnt cascades are overactivated, implicating aberrant Wnt/β-catenin signalling in FTD pathology (de la Encarnación et al., 2016; Rodríguez-Periñán et al., 2023). Although direct evidence in Lewy body dementia remains limited, the convergence of Wnt dysregulation and mTOR hyperactivation across the Alzheimer’s and other neurodegenerative spectra provides a mechanistic basis for the hypothesis that this pathway plays a wider role in neurodegenerative risk.

Moreover, mutations in LRRK2, a major genetic cause of familial and sporadic PD, directly interact with the Wnt/β-catenin pathway, modulating synaptic and neurogenic processes and linking Wnt dysregulation to Lewy body and Parkinson-related dementias (Berwick and Harvey, 2012; Harvey and Outeiro, 2019). Although direct experimental evidence in Lewy body dementia remains limited, the convergence of Wnt signalling abnormalities and mTOR hyperactivation across AD, FTD, and PD supports a shared vulnerability mechanism in neurodegeneration. However, no definitive mechanistic study to date has evaluated Wnt/mTOR interplay in dementia with Lewy Bodies or α-synucleinopathies, signalling an important gap for future research.

#### 3.2.3 Neuroinflammation and oxidative stress

Chronic low-grade inflammation and oxidative stress, characterized by elevated IL-6 and TNF-α, have been observed in ADHD and are recognized contributors to neurodegeneration (Holmes et al., 2009; Corominas-Roso et al., 2017). Impaired antioxidant defenses in ADHD may further exacerbate vulnerability to neuronal injury, synergizing with age-related processes.

Recent peer-reviewed research deepens understanding of the biological connection between ADHD and late-life dementias, including AD. A 2025 study from Switzerland proposed the first neuropathological mechanism linking ADHD to neurodegeneration (Berberat et al., 2025). This study employed quantitative susceptibility mapping (QSM) MRI and blood biomarkers (neurofilament light chain, NfL) in 61 adults (25–45 years old), showed greater iron buildup in the right precentral cortex and higher blood levels of neurofilament light chain compared to controls. Both biomarkers are recognized indicators of neuronal damage and early cognitive decline in Alzheimer’s and other dementias. The authors interpret these results as potential evidence of accelerated neurobiological aging in ADHD, mediated by iron dysregulation and oxidative stress.

Complementary findings were reported recently, where a large genetic study demonstrated that individuals with a high polygenic risk for ADHD exhibit increased risk of AD and age-related cognitive decline, mediated by dopaminergic and synaptic regulatory genes shared between both disorders (Leffa et al., 2025). However, this genetic convergence may not extend to all neurodegenerative syndromes, particularly PD. A prior genetic study analyzing nine ADHD candidate SNPs in a large European cohort found no significant association with PD after correction for multiple testing (Geissler et al., 2017).

#### 3.2.4 Psychiatric and lifestyle comorbidities

Comorbid depression, anxiety, sleep disturbances, and substance use are prevalent in adult ADHD and independently increase dementia risk (Becker et al., 2023; Levine et al., 2023). Lifestyle factors such as sedentary behavior, poor diet, and cardiovascular risk further amplify neurodegenerative susceptibility, emphasizing the need for comprehensive management strategies (Li et al., 2022; Bernacer et al., 2025).

#### 3.2.5 Cognitive reserve as a modulating factor in ADHD

ADHD has been associated with an increased risk of developing dementias beyond Parkinsonian and Lewy body-related disorders, notably AD and other age-related neurodegenerative conditions. Emerging evidence suggests that ADHD may constitute a lifelong vulnerability that reduces the brain’s capacity to tolerate neuropathology, a concept formalized as cognitive reserve (CR) (Stern, 2009; Díaz-Orueta et al., 2010).

CR refers to the brain’s ability to maintain cognitive function despite neuropathological burden. It arises from both innate capacity and life experiences, including education, occupational complexity, intellectual engagement, and lifestyle factors, which enhance neural efficiency, flexibility, and compensatory network recruitment (Stern, 2009; Díaz-Orueta et al., 2010). Structural and functional neuroimaging studies show that higher CR is associated with greater brain volume, cortical thickness, and adaptive network activation during cognitive tasks, which together confer resilience against dementia (Bartrés-Faz and Arenaza-Urquijo, 2011; Conti et al., 2021; Wei et al., 2024).

In adults with ADHD, chronic executive dysfunction, attentional inefficiency, and altered prefrontal-striatal dopaminergic signaling may reduce CR, limiting the brain’s compensatory capacity and lowering the threshold at which neuropathology manifests clinically. As noted earlier in this manuscript, neuroimaging and biomarker studies have reported features suggestive of accelerated neurobiological aging, such as iron accumulation and elevated neurofilament light chain, which may further compromise CR and contribute to heightened vulnerability to age-related cognitive decline (Berberat et al., 2025; Leffa et al., 2025).

Future research should explore whether interventions targeting CR, such as educational enrichment, cognitive training, and lifestyle modification, could mitigate the long-term dementia risk in adults with ADHD, complementing pharmacological strategies.

### 3.3 The role of stimulant treatment

Findings from a large registry-based cohort indicate that adults with ADHD who received psychostimulant medication, such as methylphenidate and amphetamine derivatives, did not show a clear increase in dementia risk (Levine et al., 2023). While the authors note that psychostimulants are cognitive enhancers hypothesized to reduce dementia risk, this remains speculative. Another recent study reported similar results (Cortes-Flores et al., 2024). Based on these results, one could hypothesize that stimulant treatment might mitigate long-term cognitive risk. Mechanistically, these treatments may normalize dopaminergic and noradrenergic tone, enhance fronto-striatal connectivity, improve cortical plasticity, and reduce oxidative stress and neuroinflammation, potentially counteracting neurodegenerative vulnerabilities associated with ADHD (Volkow et al., 2009; Véronneau-Veilleux et al., 2022; MacDonald et al., 2024). Stimulant-induced improvements in executive function, attention, and reward processing may further mitigate cognitive decline over time. However, current evidence remains largely observational, and randomized controlled trials are needed to confirm causal neuroprotective effects, determine the optimal type, dose, and duration of stimulant therapy, and clarify long-term outcomes across different dementia subtypes.

## 4 Discussion

Current longitudinal data support the hypothesis that adult ADHD is associated with increased dementia risk, particularly for non-amnestic subtypes and LBD. Convergent mechanistic evidence implicates dopaminergic dysfunction, possible Wnt/mTOR dysregulation, and chronic neuroinflammation as shared biological pathways.

Emerging research also suggests that CR may modulate this risk, with ADHD-related executive dysfunction potentially lowering the brain’s resilience to neuropathological insults. This is supported by recent large-scale data showing that ADHD symptoms, particularly inattention, persist in adults aged 50 and above and are phenotypically distinct from cognitive impairment and depression, yet independently associated with lower cognitive performance (Levine et al., 2025). Supporting this view, recent large-scale data show that ADHD symptoms—particularly inattention—persist in adults aged 50 and older and remain phenotypically distinct from cognitive impairment and depression, yet are independently associated with lower cognitive performance (Levine et al., 2025).

Further discussion should consider that ADHD-related cognitive and behavioral symptoms, such as impulsivity, attentional lapses, and poor self-regulation, may indirectly exacerbate dementia risk through adverse health behaviors, cardiovascular comorbidities, and chronic stress exposure (Li et al., 2022). Moreover, the cumulative impact of lifelong dopaminergic alterations could predispose to neurodegenerative vulnerability, emphasizing the need for integrative models that bridge psychiatric and neurodegenerative research domains.

Additionally, the chronic psychosocial stress and reduced stress resilience often observed in ADHD may contribute to neuroinflammatory cascades and hypothalamic–pituitary–adrenal axis dysregulation, both linked to accelerated cognitive aging (Peavy et al., 2009; Sharan and Vellapandian, 2024). Lifestyle risk factors, including sleep disruption, substance misuse, and sedentary habits, may further interact with genetic vulnerabilities to amplify dementia susceptibility. Understanding these bidirectional mechanisms is crucial for the design of preventive interventions (Huang et al., 2022).

From a neurobiological standpoint, dopaminergic hypofunction and fronto-striatal circuit abnormalities may represent early endophenotypes linking ADHD to later neurodegeneration (Frodl and Skokauskas, 2012). Future imaging studies combining PET and MRI modalities could clarify whether dopaminergic receptor availability and synaptic density show progressive changes predictive of cognitive decline (Seaman et al., 2019). Likewise, exploring Wnt/mTOR pathway alterations in ADHD could illuminate molecular convergence points with Alzheimer’s and Lewy body dementias (Yde Ohki et al., 2020; Grünblatt et al., 2023).

Future research should investigate whether interventions aimed at enhancing CR, such as educational enrichment, structured cognitive training, and lifestyle modifications, can attenuate long-term dementia risk in adults with ADHD. Notably, pharmacological strategies, particularly available dopaminergic stimulants, may complement non-pharmacological approaches by potentially mitigating or delaying age-related cognitive decline. However, the literature examining the impact of ADHD medications on cognitive deterioration remains very limited, highlighting the need for dedicated longitudinal studies.

While α-synuclein and amyloid/tau biomarkers have not been validated in ADHD populations, studies in neurodegenerative disorders indicate that GBA gene mutations and APOE-ε4, particularly in carriers, are associated with accelerated cognitive decline and dementia onset (Szwedo et al., 2022). These mutations lead to lysosomal dysfunction and α-synuclein accumulation (Liang et al., 2025), mechanisms also implicated in ADHD-related neurodegeneration. Investigating GBA and APOE variants in ADHD cohorts may provide insights into early biomarkers for dementia risk.

Additionally, diagnostic challenges in distinguishing ADHD from early cognitive decline in older adults remain underexplored. Clarifying the late-life ADHD phenotype is crucial for improving early identification and intervention strategies (Levine et al., 2025).

In summary, integrating genetic, neuroimaging, and neurochemical data could enhance early detection and intervention strategies for dementia in adults with ADHD.
